# Schizotypy and psychopathic tendencies interactively improve misattribution of affect in boys with conduct problems

**DOI:** 10.1007/s00787-020-01567-8

**Published:** 2020-05-31

**Authors:** Steven M. Gillespie, Mickey T. Kongerslev, Sune Bo, Ahmad M. Abu-Akel

**Affiliations:** 1 Department of Psychological Sciences, University of Liverpool, Liverpool, L69 3GB UK; 2 Department of Psychology, University of Southern Denmark, Odense, Denmark; 3 Psychiatric Research Unit, Region Zealand, Slagelse, Denmark; 4 Department of Child and Adolescent Psychiatry, Region Zealand, Roskilde, Denmark; 5 Institute of Psychology, University of Lausanne, Lausanne, Switzerland; 6 Department of Psychology, University of Haifa, Haifa, Israel

**Keywords:** Psychopathy, Schizotypal personality disorder, Conduct problems, Mentalizing, Theory of mind

## Abstract

Psychopathic tendencies are associated with difficulties in affective theory of mind (ToM), that is, in recognizing others affective mental states. In clinical and non-clinical adult samples, it has been shown that where psychopathic tendencies co-occur with schizophrenia spectrum disorders, the impairing effects of psychopathic tendencies on ToM are attenuated. These effects are yet to be examined in adolescents. We examined if the impairing effect of psychopathic tendencies on affective ToM was attenuated with increasing severity of schizotypal personality disorder (PD) in a sample of 80 incarcerated adolescent boys. We showed that the impairing effect of psychopathic tendencies on the recognition of neutral mental states, but not positive or negative mental states, was evident when the relative severity of schizotypal PD was low. However, with higher scores on both measures, we observed better performance in judging neutral mental states. The preservation of affective ToM in adolescents who show elevations in psychopathic tendencies and schizotypal PD may enable them to manipulate and extort their victims for personal gain. Our results emphasize the need to consider comorbidity in clinical case formulation when working with adolescents with conduct problems and psychopathic tendencies. More broadly, our results also suggest that the pattern of social cognitive abilities associated with co-occurring psychopathology does not always conform to an often-theorized double-dose of deficit hypothesis.

**Electronic supplementary material:**

The online version of this article (10.1007/s00787-020-01567-8) contains supplementary material, which is available to authorized users.

## Introduction

Youth with conduct problems (CP) represent a heterogeneous group who display a persistent pattern of aggressive and antisocial behavior, and who incur a considerable societal burden in terms of victimization and financial costs [1, 2]. A particular subgroup of youth with CP also present with elevated psychopathic tendencies, characterized by empathic deficits and a callous and manipulative interpersonal style [1, 3]. The presence of psychopathic tendencies in youth with CP is associated with impairments in social cognitive functioning, including in mentalizing or theory of mind (ToM) [4, 5]. However, evidence in support of this association has been mixed [6]. These inconsistent findings may reflect the presence of concurrent schizophrenia spectrum disorders (SSDs)—including schizotypal personality disorder (PD)—in antisocial youth. Schizotypal PD lies within the schizophrenia spectrum and represents one of the high-risk groups for schizophrenia [7]. The results of a meta-analysis show that up to 3% of incarcerated youth show emerging schizotypal personality disorder [8], and these symptoms are associated with impairments in social cognitive functioning [9] and increased aggressiveness [10, 11]. Although it is difficult to estimate the co-occurrence of psychopathy in adolescents with an SSD, it has been shown that up to 25% of patients with schizophrenia or schizoaffective disorder exceed the cutoff for a diagnosis of psychopathy on the Psychopathy Checklist - Revised (PLC-R) [12]. However, the extent to which psychopathic tendencies are associated with schizotypal traits in youth, and how their co-occurrence affects social cognitive functioning, remains unknown.

ToM refers to the ability to recognize and understand others’ thoughts, feelings, intentions, and beliefs, and is critical for successful social interaction [13]. As adolescents develop, substantial structural and functional changes in the brain support the development of ToM, allowing them to learn to navigate increasingly complex social relationships [14, 15]. Theory and research has divided ToM along the lines of social-perceptual and social-cognitive ToM [16], and cognitive versus affective ToM [17]. Social-perceptual ToM refers to more automatic aspects of ToM including recognition of complex mental states from facial expressions, while social-cognitive ToM includes reasoning about stories or scenarios and is commonly assessed using the False Belief Task. When divided along the lines of cognitive and affective ToM, the cognitive component allows one to infer the thoughts, intentions, and beliefs of another, while the affective component allows one to understand another’s feelings and emotions. Evidence supporting a distinction between cognitive and affective ToM is provided by a series of lesion and scanning studies that show a clear dissociation between these different abilities [17, 18]. There is also considerable evidence that these two broad components of ToM may be affected by several factors, including age [13], verbal IQ [19], and several clinical conditions that develop during adolescence, for example psychopathic tendencies [4, 20], SSDs [9, 21], and borderline PD [22].

Over the past decade, it has become increasingly clear that, like adults, children and adolescents experience various forms of personality pathology [23, 24]. In particular, the manifestation of antisocial personality pathology in youth has received considerable attention, and it is now well accepted that some youth with CP show psychopathic tendencies, characterized by a constellation of interpersonal, affective, lifestyle, and antisocial traits [1, 2]. More recently, the term ‘Limited Prosocial Emotions’ has been included as a specifier for the diagnosis of conduct disorder in the Fifth Edition of the Diagnostic and Statistical Manual of Mental Disorders (DSM-5; [25]). In developmental samples, psychopathic tendencies are generally associated with impaired ToM task performance [4, 5, 20]. The extent to which ToM abilities vary with psychopathic tendencies may be revealing about the conning and deceitful interpersonal style that is characteristic of psychopathy, and the ability to manipulate and extort others for personal gain. For example, incarcerated adolescent boys who performed better on a measure of affective ToM reported increased use of premeditated, but not reactive, aggression [20]. Understanding the impact of psychopathic tendencies on the development of affective ToM could therefore be revealing about the most appropriate methods for assessing and managing risk for violence in youth with CP.

The most frequently investigated aspect of ToM in relation to psychopathic tendencies is the ability to recognize others affective mental states, synonymous with both social-perceptual and affective ToM. This aspect of ToM has been commonly assessed using the Reading the Mind in the Eyes Test (RMET; [26]), where participants are asked to match complex social emotions shown in cropped images of the eye region with mental state descriptor words. For example, in one study of 417 children aged between 10 and 12 years, greater scores for grandiose/manipulative, callous-unemotional (CU), and impulsive/irresponsible psychopathic tendencies were each associated with impaired performance on the RMET [5]. However, when these dimensions were modelled simultaneously, the CU dimension alone was associated with poorer recognition of complex, but not basic, mental states [5]. The CU features of psychopathy were similarly related to poorer performance on both the RMET, and the Movie Assessment for Social Cognition (MASC; [27]), a more cognitively demanding top-down measure of ToM in an adolescent psychiatric inpatient sample [4].

In contrast to results in developmental samples, adult males with elevated psychopathic traits appear to show intact or even enhanced RMET task performance compared to those with lower psychopathic traits [28, 29]. The reasons for this diverging pattern of results remain unclear. One potential explanation is that as individuals with psychopathic tendencies age in to adulthood, they learn to rely on more cognitive routes for mental state recognition [30], becoming less reliant on typically impaired abilities for affective processing associated with CU traits. This hypothesis has received tentative longitudinal support, with emotion understanding at age three found to support the development of ToM at age six, in children with low, but not high, CU traits [31]. These results suggest that an alternative developmental pathway accounts for the development of ToM in children with elevated CU traits [31]. However, not all studies in adult samples have reported consistent results. When separately considering the positive, neutral, and negative subscales of the RMET, higher scores for Factor 1 (Interpersonal, Affective) of the PCL-R [32] were associated with better performance on the neutral subscale, while higher scores for Factor 2 (Lifestyle, Antisocial) were associated with poorer performance on the neutral and negative subscales [33]. The impairing effects of psychopathic tendencies on positive, neutral and negative mental state recognition have also been reported in a non-clinical adult sample [34], but these relationships have yet to be tested in developmental samples.

The relationship of schizophrenia and SSDs with ToM impairment is well established. Several meta-analyses have documented the presence of ToM difficulties in schizophrenia patients compared to healthy controls [35, 36], and among those with first-episode psychosis, at ultra-high risk for psychosis and among first-degree relatives of schizophrenia [37]. ToM impairments have also been noted in relation to schizotypal traits, referring to subclinical manifestations of the same biological and psychological factors that characterize schizophrenia and other psychoses [38]. Consistent with a diathesis-stress model of schizophrenia [38], which suggests that the disorder exists on a continuum, similar traits to those seen in patients with schizophrenia are also exhibited among individuals with schizotypy [39]. Thus, it is perhaps unsurprising that, like schizophrenia, schizotypy is associated with ToM difficulties in adults [40, 41], and in adolescents [9]. In particular, the positive symptoms of SSDs, including the presence of irrational beliefs and hallucinations, appear to be associated with a tendency to over-attribute intentionality and purpose to other individuals and events [9, 42], that is, to hypermentalize [43–45].

This tendency toward hypermentalizing has been examined in studies that tested emotion and mental state recognition in SSDs, showing a pattern of attributing emotion or intention to neutral facial expression stimuli. Patients with schizophrenia show altered salience attribution to neutral stimuli [46], and an increased tendency to judge neutral faces as negative [47, 48]. The misattribution of affect to neutral expressions in schizophrenia appears to be related to hyperactivation of the amygdala in response to neutral expressions, or hypoactivation in response to emotional expressions [48–50]. This pattern has been confirmed in a meta-analysis [51], and a similar pattern of activation for neutral versus affective social stimuli has also been observed in the posterior superior temporal sulcus (pSTS; [48, 49, 52]). Given the importance of the pSTS for recognizing the intentions of others [53, 54], hyper-engagement of this region in SSDs might represent a mechanism for false-positive perceptions of intentions (i.e., hypermentalizing) [55].

Although both psychopathic tendencies and schizotypal personality are associated with impaired ToM in developmental samples [4, 5, 9, 20], the effects of their co-occurrence on social cognitive functioning remains unclear. Given that both are associated with impairments in ToM, it may be expected that their co-occurrence would be associated with a ‘double dose of deficit’, that is, greater impairments in ToM compared to what can be observed in either disorder alone. This hypothesis has yet to be tested in a sample of adolescents, but several studies have examined the impact of SSDs with comorbid antisocial personality pathology on psychological functioning in adult men. In one study, prepulse inhibition of the startle response (that is, a reduction of the response to a startling stimulus (pulse) when this is preceded by a lower intensity stimulus (prepulse), serving as a mechanism that limits excess sensory input) was compared between violent men with comorbid dissocial PD and psychosis, violent men with psychosis alone or dissocial PD alone, and healthy controls [56]. Patients with comorbid diagnoses showed impaired prepulse inhibition compared with violent men with psychosis alone and a healthy control group, and this finding was interpreted to reflect a double dose of deficit [56]. However, performance in the comorbid group was similar to that of dissocial PD patients, and there was a negative effect of PCL-R Factor 2 (Lifestyle, Antisocial) scores on prepulse inhibition [56]. As such, these differences may more simply reflect the presence of antisocial personality pathology more generally, rather than the effects of comorbid diagnoses.

A potential ‘double dose of deficit’ has also been noted in a systematic review, with lower IQ and poorer face affect recognition reported for patients with comorbid antisocial PD and schizophrenia, compared with schizophrenia alone [57]. However, the converse pattern has also been reported; in a neuroimaging study it was found that healthy controls outperformed patients with schizophrenia, but not violent offenders with schizophrenia and comorbid antisocial PD, during an affective ToM task [58]. The findings reviewed here highlight important heterogeneity among violent offenders with SSDs, but it is important to note that psychopathy is distinguishable from antisocial and dissocial personalities by the presence of affective and interpersonal features [59], and as such these results may not be generalizable to psychopathy.

In contrast to findings for schizophrenia with comorbid antisocial personality pathology, better social cognitive abilities have been reported for co-occurring SSDs and psychopathic tendencies [60–62]. In a recent study, it was found that psychopathic tendencies in a European schizophrenia patient sample were associated with lower metacognitive abilities [60]. However, this relationship changed for patients with more extreme scores for psychopathy, with increasing psychopathic tendencies associated with better metacognitive abilities. Notably, the point at which this relationship changed was close to the cut-off score for diagnosing psychopathy in Europe using the PCL-R (≥ 25) [32, 60]. In a separate study with a non-clinical sample, psychopathic tendencies were associated with fewer errors in mentalizing among participants reporting more frequent positive psychotic experiences, but not among those reporting few positive psychotic experiences [61].

The findings reported above have implications for the assessment and management of adult forensic samples, highlighting the need for comprehensive assessments that measure multiple domains of psychopathology. However, it is also important to understand the effects of psychopathic tendencies that co-occur with SSDs in adolescents, where appropriate assessment and management could have implications for interventions targeting social-cognition and aggressive and antisocial behavior. For example, although adolescents referred for psychotic experiences would not usually be assessed for psychopathic tendencies, the presence of these traits may impact in important ways on social cognitive abilities. Moreover, interventions aimed at increasing affect recognition have been recommended for youth with psychopathic tendencies [63], but such interventions may be contraindicated for youth with comorbid SSDs.

In the present study, we aimed to examine the interactive effects of clinically assessed, dimensional ratings of psychopathic tendencies, and symptoms of schizotypal PD, on affective ToM in a sample of incarcerated adolescent boys. We predicted that while psychopathic tendencies and symptoms of schizotypal PD would be independently associated with impairments in affective ToM, their interaction would be associated with improved performance. Given that SSDs appear to be associated with a pattern of hypermentalizing about others mental states, characterized by the misattribution of emotion to others neutral expressions, we predicted that schizotypal PD would be most strongly associated with an increased number of errors for neutral stimuli. In contrast, psychopathic tendencies appear to be associated with a reduced tendency to misattribute affect to neutral expressions (i.e., a reduced tendency to hypermentalize). Therefore, we predicted that better performance for co-occurring psychopathic tendencies and symptoms of schizotypal PD would be most pronounced for neutral mental states.

We also aimed to examine these relationships while controlling for a number of important confounding factors, including borderline PD and verbal IQ. Rates of borderline PD are elevated among adolescents with conduct problems, and are associated with increased aggressiveness and either enhanced [64] or impaired [65] affective ToM. Higher verbal IQ is also related to enhanced affective ToM [19], while a complex relationship between IQ and psychopathic tendencies has been reported [66]. The present study is important because it evaluates the effects of co-occurring traits during a critical period in the development of social behavior and mental illness onset [67, 68]; the predicted pattern of results would stress the importance of assessing both psychopathic tendencies and SSDs among youth with conduct problems, and understanding their relationship with social cognition.

## Method

### Participants

A sample of 80 incarcerated adolescent boys was recruited from three secure institutions for juvenile offenders in Denmark (see Table 1 for sample demographic and clinical characteristics). The initial sample consisted of 127 juvenile offenders who were assessed for eligibility. Inclusion criteria were being male, between 15 and 18 years (inclusive; this is the minimum and maximum age range in which minors can be judicially incarcerated in Denmark), sufficiently fluent in Danish, and willing and able to give informed consent. A total of 47 participants were excluded from the final sample: 27 refused to participate, 15 did not meet inclusion criteria (4 were girls; 3 were under the age of 15; 2 were unable to give informed consent due to acutely severe mental disorder; 6 did not understand Danish sufficiently), and 5 met exclusion criteria (1 was described in files as having severe mental retardation (sic); 2 were intoxicated on the day of assessment; and 2 were actively psychotic on the day of assessment).

**Table 1:** Sample characteristics (*n* = 80)

Variable		Category/description	Mean	SD
Age			16.50	0.75
Vocabulary subtest of the WISC-III/WAIS-III^a^			8.51	1.14
Psychopathy Checklist: Youth Version (PCL-YV)			20.58	8.16
Schizotypal Personality Disorder (SCID-II^b^ dimensional)			0.73	1.36
Borderline Personality Disorder (SCID-II^b^ dimensional)			2.64	1.78
Reading the Mind in the Eyes Test (RMET)			23.38	4.07
		RMET Positive Subscale	4.51	1.53
		RMET Negative Subscale	7.75	2.08
		RMET Neutral Subscale	11.11	2.04

		N		%
Ethnicity				
	Danish	39	48.75	
	Immigrant	16	20	
	Descendant	25	31.25	
Present education level				
	In high school	5	6.25	
	Technical school apprenticeship	6	7.50	
	In elementary school	15	18.75	
	Municipal education project	18	22.50	
	None	36	45.00	
Reason for placement in secure institution				
	Remand	67	83.75	
	Sentenced	13	16.25	
Previous placement in secure institution		35	43.75	
Most severe index offence (ordered by frequency)				
	Robbery (including mugging)	49	61.25	
	Assaults	18	22.50	
	Theft	6	7.50	
	Murder/attempted murder	3	3.75	
	Major driving offences	2	2.50	
	Sex offences	1	1.25	
	Possession of weapons	1	1.25	
Personality disorders (SCID-II^b^ diagnosis)				
	Cluster A (paranoid, schizotypal, schizoid)	16	20.00	
	Cluster B (histrionic, narcissistic, borderline, antisocial)	52	65.00	
	Cluster C (avoidant, dependent, possessive-compulsive)	2	2.50	
Any DSM-IV 10 personality disorders		52	65.00	
Mental disorders (K-SADS-PL^c^)				
	Mood disorders	6	7.50	
	Schizophrenia and other psychotic disorders	1	1.25	
	Anxiety disorders	14	17.50	
	ADHD	18	22.50	
	Conduct disorder	61	76.25	
	Oppositional defiant disorder	31	38.75	
	Substance abuse	31	38.75	
	Tic disorders	2	2.50	

^a^Proxy scores for verbal IQ were obtained using the vocabulary subtest of the Wechsler Intelligence Scale for Children-Third Edition (WISC-III) in boys < 17 years, and the Wechsler Adult Intelligence Scale-Third Edition (WAIS-III) in boys ≥ 17 years

^b^*SCID-II* Structured Clinical Interview for DSM Disorders Axis II Disorders

^c^*K-SADS-PL* Schedule for Affective Disorders and Schizophrenia for School age Children-Present and Lifetime Version

### Assessment instruments

#### Psychopathic tendencies

Psychopathic tendencies were assessed using the widely used Psychopathy Checklist: Youth Version (PCL-YV; [3]). The PCL:YV is a clinical construct rating scale for use with youth aged 12–18, and comprises 20 items tapping the Affective, Interpersonal, Lifestyle, and Antisocial features of psychopathy. The instrument yields a dimensional total score ranging from 0 to 40. Acceptable psychometric properties have been reported for the PCL:YV [69]. In this study, interrater agreement for the PCL:YV total score in a randomly selected subset of our sample (*n* = 20) was excellent (intraclass correlation coefficient (ICC) = 0.91) [70].

#### Affective theory of mind

The RMET [26] was used to assess the recognition of others affective mental states. The RMET examines participants’ ability to recognize emotions in others based on 36 photographs cropped to show the eyes of emotionally expressive faces. Participants are asked to make a forced choice as to which word from a choice of four best matches what the person in the photograph is thinking or feeling. For each photograph, three of the four words are distractor items, while one of the words represents the correct answer. The 36 items were subdivided in to images depicting negative (*n* = 12), neutral (*n* = 16), and positive (*n* = 8) emotional states, using the classification procedure derived by Harkness et al. [71].

#### Additional measures

To assess for symptoms of PD, we used the Structured Clinical Interview for DSM-IV Axis II Personality Disorders (SCID-II; [72]). The SCID-II is a semi-structured interview consisting of 119 sets of questions; additional questions are included to assess antisocial PD and conduct disorder prior to age 15. The questions correspond to diagnostic criteria for the respective DSM-IV/DSM-5 PD, and are scored as either 1 = absent; 2 = subthreshold; 3 = true; or ‘?’ = inadequate information. Each PD was dimensionally scored by summing the number of diagnostic criteria marked ‘true’. The number of available diagnostic criteria varied between seven and nine for each specific PD, with both schizotypal PD and borderline PD scored dimensionally on a scale from zero to nine. The SCID-II has acceptable psychometric properties in adolescent populations [24, 73]. Interrater agreement in a randomly selected subset of the sample (*n* = 40) was good to excellent, with Kappa values for agreement in diagnosis ranging from 0.73 to 0.90 across different PD, with ICC for dimensional agreement of diagnostic criteria ranging from 0.71 to 0.90 across different PD [74].

To assess for concurrent psychiatric diagnoses, we used the Schedule for Affective Disorders and Schizophrenia for School age Children-Present and Lifetime Version (K-SADS-PL; [75]). The K-SADS-PL is a semi-structured interview for assessment of current and past psychopathology in children aged 6–18 years, according to DSM-IV criteria. In this study, we only assessed for current psychopathology, and did not include family members as informants. Interrater agreement in a subset of the sample was excellent, with kappa values for categorical agreement and intraclass correlations (ICC) for dimensional agreement of symptoms ranging from 0.77 to 0.86.

Finally, we used the vocabulary subtest of the Wechsler Intelligence Scale for Children-Third Edition (WISC-III; [76]) in boys < 17 years, and the Wechsler Adult Intelligence Scale-Third Edition (WAIS-III; [77]) in boys ≥ 16 years as a proxy for verbal intelligence. Inter-rater agreement in a subset of the sample (*n* = 20) was excellent for this subtest (ICC = 0.82) [70].

### Procedure

To minimize risk of coercion, members of staff at the secure institutions—who were independent of the research project—approached eligible participants and provided information about the study. The researcher later met with potential participants who were interested in taking part and provided further details about the study, including a written description. It was emphasized throughout that participation was voluntary, and that the decision not to take part would have no implications for the individual within the secure sites or in the judicial system. All participants provided written informed consent. The second author performed all assessments in a quiet room at the secure sites. All measurers were completed as part of a longer test battery for the purposes of research and were not completed as part of regular assessments. Depending on progress, measures were typically completed over two to three sessions, taking between 4 and 8 h in total. Participants were not offered any reward for their participation.

Assessments were audio recorded to allow for assessment of interrater agreement. Following the completion of data collection, an experienced clinical psychologist (the third author) read through file information and listened to randomly selected recordings of the PCL:YV, SCID-II, K-SADS-PL, and WISC-III/WAIS-III assessments, providing independent ratings so that interrater agreement could be established.

### Analytic approach

The main analyses were conducted using generalized linear regression models (GLMs). These were used to examine the association of psychopathy, schizotypal PD severity, and their interaction on affective ToM (measured in terms of the RMET total score, and its negative, positive and neutral subscales). All GLMs were conducted with robust estimators, which allow for the treatment of non-normally distributed data and guards against outliers being unduly influential [78]. For all GLMs, we report the Chi-squared Omnibus test statistic, which is a likelihood-ratio Chi-square test of the model (with predictors) versus the null, intercept model. Parameter estimates of the model are interpretable if the Omnibus test is significant (*p* < 0.05). Effect sizes for the GLMs were calculated in terms of Pseudo *R*^2^ using the following formula:

$$1 - \frac{{\text{ Deviance}}}{{{\text{Null}}\;{\text{Deviance}}}}$$

Because performance on the RMET has also been found to vary with age [13], verbal IQ [19], and borderline PD [64, 65], associations were examined while controlling for age, proxy scores for verbal IQ, and severity of borderline PD. Where applicable, False Discovery Rate (FDR) correction was applied to control for multiple testing [79]. Significant interactions were probed with the Johnson–Neyman method using R Version 3.3.3 [80]. This method provides a ‘high-resolution picture’ of the interaction by estimating the value(s) of one predictor, at which the other predictor (or the moderator) has a significant effect on the outcome measure (i.e., the significance zone). This is established by identifying the precise value(s) along the continuum of one predictor for which the regression slopes of the other predictor are estimated to be significantly different from zero.

## Results

Sample characteristics, including participants’ scores on the PCL:YV, RMET and subscales, and severity of schizotypal and borderline PD are summarized in Table 1. About half of the participants (49%) were Danish, and the remaining 51% of participants were either immigrants or descendants, primarily from the Middle East, Northern Africa, or Europe. Most of the participants were remanded, and the majority of participants index offense involved violence (e.g., robbery (including mugging), assault, murder/attempted murder), and approximately half had lost contact with the educational system prior to incarceration. We note, as shown in Table 1, that although the rates of PD and other psychiatric disorders were high, they are nonetheless comparable with those reported in other studies of incarcerated youth [81].

Table 2 shows the inter-correlations of age, proxy scores for verbal IQ, RMET total and its positive, negative, and neutral subscales, PCL:YV total scores, and severity of schizotypal and borderline PD. Higher PCL:YV total and schizotypal PD severity scores were associated with poorer performance on both RMET total and its neutral subscale. Severity of borderline PD was associated with poorer performance on the neutral subscale of the RMET. As expected, higher proxy verbal IQ was associated with better performance on RMET total, and its negative and neutral subscales. The plot in Online Resource 1 shows that increasing PCL:YV scores were exponentially associated with greater severity of schizotypal PD.

**Table 2:** Inter-correlations between measures (*n* = 80)

Variable	1	2	3	4	5	6	7	8	9
1. Age									
2. Proxy verbal IQ	0.25*								
3. RMET^a^ total	0.13	0.32**							
4. RMET^a^ negative	0.16	0.25*	0.76***						
5. RMET^a^ positive	0.05	0.21	0.61***	0.22					
6. RMET^a^ neutral	0.06	0.23*	0.76***	0.34**	0.24*				
7. PCL:YV^b^ total	0.09	− 0.08	− 0.32**	− 0.16	− 0.13	− 0.39***			
8. Schizotypal PD (SCID-II^c^ dimensional)	− 0.11	0.08	− 0.30**	− 0.14	− 0.18	− 0.31**	0.42***		
9. Borderline PD (SCID-II^c^ dimensional)	0.11	− 0.13	− 0.17	0.04	− 0.08	− 0.32**	0.37***	0.37***	
Range	15–18 (years)	6–11	15–31	3–11	1–8	7–15	3–35	0–7	0–7
Skewness	− 0.66	− 0.27	− 0.21	− 0.19	0.05	− 0.33	− 0.11	2.99	0.56
Kurtosis	− 0.35	− 0.60	− 0.75	− 0.46	− 0.43	− 0.53	− 0.89	9.96	− 0.68

*p < 0.05, **p < 0.01, ***p < 0.001

^a^*RMET* Reading the Mind in the Eyes Test

^b^*PCL:YV* Psychopathy Checklist: Youth Version

^c^*SCID-II* Structured Clinical Interview for DSM Disorders Axis II Disorders

Next, as seen from Table 3, the GLMs yielded significant overall models, as suggested by the omnibus tests, for the RMET total (*χ*^2^ = 22.78, *df* = 6, *p*_FDR-cor_ = 0.002, Pseudo *R*^2^ = 0.248), and the RMET neutral subscale (*χ*^2^ = 25.89, *df* = 6, *p*_FDR-cor_ < 0.001, Pseudo *R*^2^ = 0.274), explaining 24.8% and 27.4% of the variance, respectively. The parameter estimates of the RMET total model revealed a significant positive association for proxy verbal IQ, and a significant negative association for PCL:YV total scores. The parameter estimates for age, borderline PD severity, schizotypal PD severity, and for the interaction of PCL:YV total scores with schizotypal PD severity were non-significant. The parameter estimates of the RMET neutral subscale model revealed significant negative associations for both PCL:YV total scores and severity of schizotypal PD. However, these associations were qualified by a positive PCL:YV total × schizotypal PD interaction. RMET neutral was marginally associated with proxy verbal IQ (*p* = 0.051) and non-significantly with age and borderline PD severity. The omnibus tests were non-significant for the RMET negative (*χ*^2^ = 11.51, *df* = 6, *p*_FDR-cor_ = 0.098, Pseudo *R*^2^ = 0.134), and the RMET positive (*χ*^2^ = 9.93, *df* = 6, *p*_FDR-cor_ = 0.128, Pseudo *R*^2^ = 0.117) subscales.

**Table 3:** Parameter estimates of the RMET total and the RMET neutral subscale models (*n* = 80)

Variable	*Β*	SE	Wald *χ*^2^	*df*	*p* value
RMET^a^ total (*χ*^2^ = 22.78, *df* = 6, *p*_FDR-cor_ = 0.002, Pseudo *R*^2^ = 0.248)					
Age	0.147	0.510	0.083	1	0.773
Proxy verbal IQ	1.144	0.353	10.48	1	**0.001**
Borderline PD severity	0.132	0.251	0.28	1	0.599
Schizotypal PD severity	− 1.975	1.599	1.52	1	0.217
PCL:YV^b^ total	− 0.125	0.054	5.34	1	**0.021**
PCL:YV^b^ total × schizotypal PD severity	0.046	0.056	0.67	1	0.413
RMET^a^ neutral subscale (*χ*^2^ = 25.89, *df* = 6, *p*_FDR-cor_ < 0.001, Pseudo *R*^2^ = 0.274)					
Age	− 0.001	0.216	0.000	1	0.998
Proxy verbal IQ	0.352	0.180	3.80	1	0.051
Borderline PD severity	− 0.107	0.104	1.05	1	0.305
Schizotypal PD severity	− 1.851	0.670	7.06	1	**0.008**
PCL:YV^b^ total	− 0.086	0.027	10.10	1	**0.001**
PCL:YV^b^ total × schizotypal PD severity	0.059	0.025	5.58	1	**0.018**

Bold values are significant (*p* < 0.05)

^a^*RMET* Reading the Mind in the Eyes Test total score

^b^*PCL:YV* Psychopathy Checklist: Youth Version; *PD* Personality Disorder (dimensionally assessed)

**Fig. 1:**
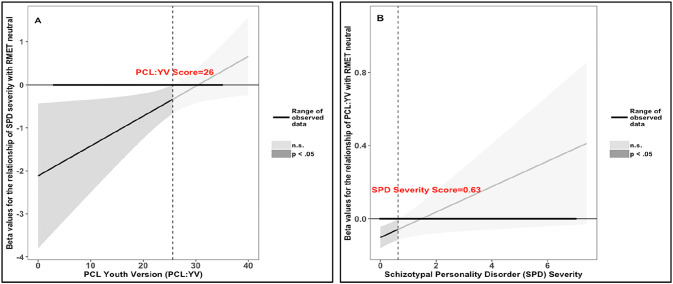
The interactive association of schizotypal personality disorder (SPD) severity and total Psychopathy Checklist: Youth Version (PCL:YV) scores with RMET neutral subscale scores. **a** displays the association (*β* weights) of SPD severity with participants’ performance on the neutral subscale of the RMET along the range of the PCL-YV total scores. The *β* values for the association of SPD severity with RMET neutral become less negative with increasing psychopathic tendencies, and cease to be significant when the PCL:YV score ≥ 26. **b** Displays the association (*β* weights) of PCL:YV scores with participants’ performance on the neutral subscale of the RMET along the range of the SPD severity. The *β* values for the association of PCL:YV scores with RMET neutral become less negative with increasing SPD severity, and cease to be significant when the SPD severity ≥ 0.63. Shaded areas represent the 95% Confidence Interval of the slopes lines (*β* weights). Dark grey areas represent the zone of significant associations (*p* < 0.05). Light grey areas represent the zone of non-significant associations

The probe of the positive PCL:YV total × schizotypal PD interaction on the scores of the RMET neutral subscale revealed that the negative association of schizotypal PD severity with correctly identifying neutral expressions is arrested at extreme values of the PCL:YV. Specifically, Fig. 1a shows that as PCL:YV scores increase, the *β* values for the association of schizotypal PD severity with RMET neutral become less negative, and the effect remains significant for scores up to 26 on the PCL:YV. For PCL:YV scores exceeding this point, the relationship of schizotypal PD severity with RMET neutral ceases to be significant. Thus, for participants whose PCL:YV scores exceeded the cut-off point for diagnosing psychopathy on the PCL:YV, the impairing effect of schizotypal PD severity on neutral mental state recognition was close to zero. In addition, the negative association of the PCL:YV levels on correctly identifying neutral expressions is only significant when the severity of schizotypal PD is virtually absent (see Fig. 1b).

In Online Resource 2, we examined which of the four facets of the PCL:YV might be driving the positive interaction between PCL:YV total scores and schizotypal PD for the RMET neutral subscale. Results suggested that the interaction was driven by the Affective and the Antisocial facets of the PCL:YV, but not the Interpersonal or the Lifestyle facets.

## Discussion

In this study, we investigated if psychopathic tendencies (PCL:YV), severity of schizotypal PD (SCID-II), and their interaction, were associated with affective ToM (RMET) in boys with CP. Using separate GLMs, we found that after controlling for proxy verbal IQ scores and severity of borderline PD, both psychopathic tendencies and schizotypal PD were associated with impaired recognition of neutral, but not positive or negative, mental states. Intriguingly, however, their two-way interaction was associated with better performance on the neutral subscale. The beneficial effects of the two-way interaction suggest that although both SSDs and psychopathic tendencies alone are associated with impairments in recognizing others’ affective mental states, there may be differences in the underlying pattern of errors that characterizes each disorder.

When looking at the overall pattern of performance on the RMET, we found a positive effect of proxy verbal IQ scores and a negative effect of psychopathic tendencies. These findings are consistent with the results of several studies showing that greater IQ is associated with improved performance on the RMET, and that IQ represents a vital component for understanding and recognizing others affective mental states [19]. In contrast, it has been reported that the presence of elevated psychopathic tendencies in children and adolescents is associated with impairments in affective ToM [4, 5, 20]. The findings reported here are therefore consistent with earlier work in adolescent samples. Severity of both schizotypal and borderline PD was unrelated to overall performance on the RMET. These findings are in contrast to earlier results showing that both schizotypal [9] and borderline [64, 65] PD are associated with affective ToM.

Next, we examined the effects for the positive, neutral, and negative subscales of the RMET individually. Although models for the positive and negative subscales were non-significant, we showed that both psychopathic tendencies and severity of schizotypal PD had an impairing effect on the recognition of neutral mental states when the relative severity of the other dimension was low (see Fig. 1). Severity of borderline PD was unrelated to performance on the neutral subscale, and greater IQ was marginally related. Earlier studies in adult community [34] and offender [33] samples have been equivocal about the effects of psychopathic tendencies on RMET neutral mental state recognition. However, there is considerable evidence to support our finding that schizotypal personality and SSDs are associated with impaired classification of others’ neutral expressions as neutral [47, 48].

Intriguingly, the significant positive two-way interaction of psychopathic tendencies with severity of schizotypal PD indicated that higher scores on both measures together were associated with better categorization of neutral mental states. Results reported in Online Resource 2 of this report suggest that this interaction may be driven by the Affective and Antisocial facets of the PCL:YV, which were both associated with improved neutral mental state recognition at higher severity of schizotypal PD. Although seemingly paradoxical, these results are consistent with earlier work showing that psychopathic tendencies were associated with improvements in metacognitive abilities among patients with a diagnosis of schizophrenia, but only among those who scored above the cutoff for a diagnosis of psychopathy on the PCL-R [60]. An inverse association of psychopathic tendencies with metacognition was found among those who scored below the cutoff [60]. Remarkably, here too, better performance was observed at a score of 26 on the PCL:YV, which is around the recommended cut-off score (≥ 25) for the PCL:YV in Europe [70]. Similarly, when examining the broader phenotypic constructs of psychopathy and positive psychotic experiences in a young adult sample, it was reported that elevated scores on both dimensions were related to better ToM task performance [61]. The results reported here are the first to show evidence for a similar pattern of results in a sample of incarcerated adolescent boys.

In an attempt to shed light on the mechanism underlying this pattern of results, it is important to consider the mechanisms that underlie impairments in ToM task performance in psychopathy and SSDs. It has been reported that SSDs are associated with a pattern of hypermentalizing that is evident, for example, in the tendency to attribute affect to neutral face stimuli [47, 48]. On the other hand, the underlying mechanism associated with ToM impairment in psychopathy is not well known. One study showed that the callous and uncaring features of psychopathy may also be related to excessive ToM or hypermentalizing in adolescents performing a top-down measure of ToM [4]. However, this pattern of results was described as surprising, especially in light of findings that hypermentalizing is most characteristic of disorders that are associated with high affect, including depression and borderline PD [65]. In contrast, Sandvik et al. [33] showed that the Affective and Interpersonal features of psychopathy among adult offenders were associated with better neutral mental state recognition, indicative that psychopathy is not associated with hypermentalizing. To better understand our findings and their potential application, it is important for future research to investigate the underlying mechanism of ToM impairments in psychopathic personality (e.g., the tendency to hypermentalize versus hypomentalize).

Our findings may have implications for the assessment of youth with CP. One clear implication is that to fully understand an individual’s social cognitive abilities, it is important to consider the presence of co-occurring traits or comorbid conditions. Thus, we would recommend that youth with a suspected SSD and CP should also be assessed for the presence of elevated psychopathic tendencies. Understanding the presence of these traits may allow for a more complete formulation of an individual’s needs and difficulties. In relation to treatment, the findings of the current study would contraindicate the use of interventions aimed at enhancing mentalizing about others affective states in youth with CP and co-occurring SSDs and psychopathic tendencies. Instead, the aim of early intervention with this group should instead be to help them to use their relatively enhanced ToM abilities for more prosocial means [20].

A crucial corollary of this is that our findings may also have implications for understanding the aggressive behaviors associated with co-occurring psychopathic tendencies and SSDs. For example, better ToM abilities may be associated with a more cunning and deceitful interpersonal style in psychopathy and have been linked with a greater incidence of premeditated aggression among incarcerated adolescent boys [20]. Thus, it may be predicted that the aggressive behaviors of those with elevated psychopathic tendencies and SSDs would be more instrumental compared with those who score relatively more highly on one measure compared with the other. Tentative evidence for this hypothesis has been reviewed [82], although as yet, a direct test of the interactive effects of psychopathy and SSDs on reactive and proactive aggression has not been reported.

Our results need to be interpreted in light of several limitations. The results reported here are based on a moderate sized sample of incarcerated adolescent boys, and our findings cannot be generalized to either adolescent girls or non-incarcerated boys. This importance of replicating our findings in different samples is highlighted by evidence which shows that the psychopathy construct may manifest differently for boys and girls [83], and that girls show generally elevated levels of ToM abilities compared to their male counterparts [26]. The cross-sectional nature of our design also precludes firm inferences about causality. In relation to the measure of ToM, some studies have reported adequate performance on the RMET in relation to borderline PD, suggesting that this task may not be sensitive to the complexities of ToM dysfunction in personality disordered samples [64]. We therefore recommend that future research should aim to examine the effects of these co-occurring traits using other experimental ToM tasks, for example, the Director Task [84], and the Movie Assessment of Social Cognition [27]. Finally, the specificity of these results to psychopathy and SSDs remains unclear, particularly when the pattern of results observed here is reminiscent of the better performance observed for probabilistic reasoning in individuals with obsessive compulsive and delusional disorders [85], and for ToM and social functioning in individuals with co-occurring autism and SSDs [86, 87]. If, as seems possible, the co-occurrence of certain psychopathologies can moderate ToM impairments, the simultaneous assessment of different dimensions within clinical settings should be prioritized. This would be a necessary step for building multidimensional models of psychopathology.

Our study shows that reports of better social-cognitive abilities associated with co-occurring psychopathic tendencies and SSDs in adults also apply to a sample of incarcerated adolescent boys. In particular, we found that increasing severity of schizotypal PD was associated with poorer affective ToM, but only among those scoring below the cut-off score for diagnosing psychopathy using the PCL:YV. In contrast, the relationship of severity of schizotypal PD with affective ToM was no longer significant for youth who scored above the cut-off score on the PCL:YV. Our results underscore the importance of comprehensive assessment in youth with CP, and suggest that the pattern of social cognitive abilities associated with co-occurring psychopathology does not always conform to an oft-theorized double-dose of deficit hypothesis.

## Electronic supplementary material

Below is the link to the electronic supplementary material.

Supplementary file1 (DOCX 87 kb)

Supplementary file2 (DOCX 167 kb)
