# Supplementary material for: Schizotypy and psychopathic tendencies interactively improve misattribution of affect in boys with conduct problems
Source: Eur Child Adolesc Psychiatry. 2020 May 31;30(6):885–97. doi: 10.1007/s00787-020-01567-8 (PMC8140966; doi:10.1007/s00787-020-01567-8)
Supplement: Supplementary file 1 — Supplementary file1 (DOCX 87 kb) [file 787_2020_1567_MOESM1_ESM.docx]

Schizotypy and psychopathic tendencies interactively improve misattribution of affect in

boys with conduct problems

European Child & Adolescent Psychiatry

Steven M. Gillespie^1*^, Mickey T. Kongerslev^2,3^,

Sune Bo^3,4^, Ahmad M. Abu-Akel^5,6^

^1^Department of Psychological Sciences, University of Liverpool, Liverpool, U.K.

ORCID: 0000-0001-7789-5381

steven.gillespie@liv.ac.uk

^2^Department of Psychology, University of Southern Denmark, Odense, Denmark

^3^Psychiatric Research Unit, Region Zealand, Slagelse, Denmark

^4^Department of Child and Adolescent Psychiatry, Region Zealand, Roskilde, Denmark

^5^Institute of Psychology, University of Lausanne, Lausanne, Switzerland

^6^Department of Psychology, University of Haifa, Haifa, Israel

Electronic Supplementary Material 1


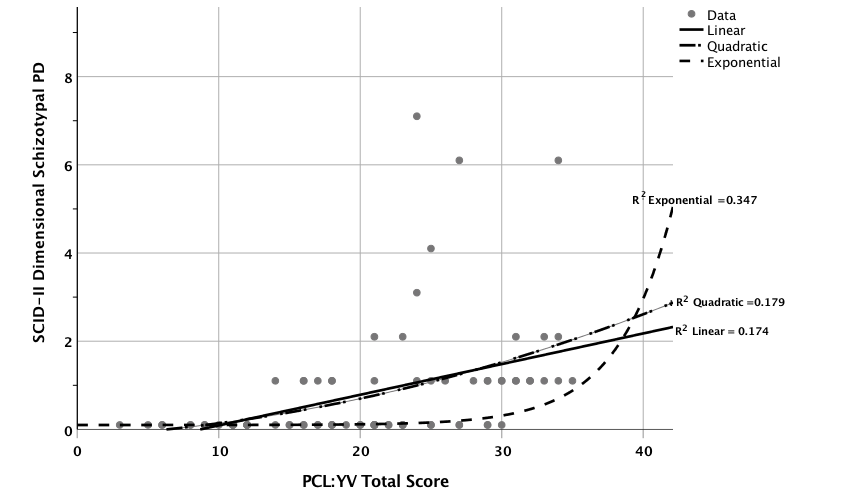


**Fig. S1** Plot showing linear, quadratic and exponential relationships of Psychopathy Checklist: Youth Version (PCL:YV) scores with schizotypal personality disorder severity. All linear (*F*(1,78) = 16.45, *p* < .001, *R^2^* = .174), quadratic (*F*(2,77) = 8.38, *p* = .001, *R^2^* = .179) and exponential (*F*(1,78) = 41.47, *p* < .001, *R^2^* = .347) models were significant. The exponential model provided the best fit to the data, explaining 34.7% of the variance.
