# Supplementary material for: Schizotypy and psychopathic tendencies interactively improve misattribution of affect in boys with conduct problems
Source: Eur Child Adolesc Psychiatry. 2020 May 31;30(6):885–97. doi: 10.1007/s00787-020-01567-8 (PMC8140966; doi:10.1007/s00787-020-01567-8)
Supplement: Supplementary file 2 — Supplementary file2 (DOCX 167 kb) [file 787_2020_1567_MOESM2_ESM.docx]

Schizotypy and psychopathic tendencies interactively improve misattribution of affect in

boys with conduct problems

European Child & Adolescent Psychiatry

Steven M. Gillespie^1*^, Mickey T. Kongerslev^2,3^,

Sune Bo^3,4^, Ahmad M. Abu-Akel^5,6^

^1^Department of Psychological Sciences, University of Liverpool, Liverpool, U.K.

ORCID: 0000-0001-7789-5381

steven.gillespie@liv.ac.uk

^2^Department of Psychology, University of Southern Denmark, Odense, Denmark

^3^Psychiatric Research Unit, Region Zealand, Slagelse, Denmark

^4^Department of Child and Adolescent Psychiatry, Region Zealand, Roskilde, Denmark

^5^Institute of Psychology, University of Lausanne, Lausanne, Switzerland

^6^Department of Psychology, University of Haifa, Haifa, Israel

Electronic Supplementary Material 2

To examine which of the Psychopathy Checklist: Youth Version (PCL:YV) facets might be driving the positive interaction we observed between the PCL:YV total and schizotypal personality disorder (SPD) severity for the RMET neutral subscale, we conducted four separate GLMs including the Interpersonal, Affective, Lifestyle, and Antisocial features of the PCL:YV. As can be seen from Table S1, all omnibus tests were significant. Notably, however, the parameter estimates indicate that the interaction between the PCL:YV total score and the SPD severity score is driven by the Affective and the Antisocial facets. We probe each of these interactions in turn.

Table S1. Parameter estimates of the RMET neutral subscale models with each of the four facets of the PCL:YV (*N*=80)

| Variable | β | SE | Waldχ^2^ | df | p-value |
| --- | --- | --- | --- | --- | --- |
| *PCL:YV Interpersonal Model (χ^2^ = 18.58, df = 6, p_FDR-cor_ = 0.005, Pseudo R^2^ = 0.207)* | | | | | |
| Age | -0.018 | 0.246 | 0.01 | 1 | 0.941 |
| Proxy verbal IQ | 0.422 | 0.198 | 4.54 | 1 | **0.033** |
| Borderline PD Severity | -0.235 | 0.107 | 4.85 | 1 | **0.028** |
| Schizotypal PD Severity | -0.359 | 0.339 | 1.12 | 1 | 0.290 |
| Interpersonal | -0.093 | 0.117 | 0.63 | 1 | 0.428 |
| Interpersonal x Schizotypal PD Severity | -0.005 | 0.080 | 0.00 | 1 | 0.953 |
| *PCL:YV Affective Model (χ^2^ = 30.97, df = 6, p_FDR-cor_ < 0.001, Pseudo R^2^ = 0.321)* | | | | | |
| Age | -0.057 | 0.209 | 0.08 | 1 | 0.785 |
| Proxy verbal IQ | 0.269 | 0.196 | 1.88 | 1 | 0.170 |
| Borderline PD Severity | -0.157 | 0.096 | 2.67 | 1 | 0.102 |
| Schizotypal PD Severity | -2.335 | 0.774 | 9.09 | 1 | **0.003** |
| Affective | -0.320 | 0.096 | 11.13 | 1 | **0.001** |
| Affective x Schizotypal PD Severity | 0.296 | 0.105 | 7.98 | 1 | **0.005** |
| *PCL:YV Lifestyle Model (χ^2^ = 21.15, df = 6, p_FDR-cor_ = 0.002, Pseudo R^2^ = 0.232)* | | | | | |
| Age | 0.069 | 0.221 | 0.10 | 1 | .753 |
| Proxy verbal IQ | 0.315 | 0.185 | 2.89 | 1 | .089 |
| Borderline PD Severity | -0.153 | 0.111 | 1.90 | 1 | .168 |
| Schizotypal PD Severity | -1.425 | 1.001 | 2.03 | 1 | .155 |
| Lifestyle | -0.199 | 0.093 | 4.56 | 1 | **.033** |
| Lifestyle x Schizotypal PD Severity | 0.151 | 0.123 | 1.49 | 1 | .222 |
| *PCL:YV Antisocial Model (χ^2^ = 22.21, df = 6, p_FDR-cor_ = 0.002, Pseudo R^2^ = 0.242)* | | | | | |
| Age | -0.057 | 0.232 | 0.06 | 1 | .808 |
| Proxy verbal IQ | 0.367 | 0.177 | 4.29 | 1 | **0.038** |
| Borderline PD Severity | -0.115 | 0.111 | 1.08 | 1 | .300 |
| Schizotypal PD Severity | -1.273 | 0.403 | 9.98 | 1 | **0.002** |
| Antisocial | -0.180 | 0.085 | 4.55 | 1 | **0.033** |
| Antisocial x Schizotypal PD Severity | 0.142 | 0.060 | 7.76 | 1 | **0.005** |

Note: RMET = Reading the Mind in the Eyes Test total score; PCL:YV = Psychopathy Checklist: Youth Version; PD = Personality Disorder (dimensionally assessed)

The probe of the positive PCL:YV Affective x SPD severity interaction revealed that the negative association of SPD severity with correctly identifying neutral expressions reduces with increasing scores on the Affective facet of the PCL:YV, and is arrested when PCL:YV Affective scores exceed 6.54 (see Fig. 1SA). Thus, for participants whose PCL:YV Affective scores exceeded 6.54, the impairing effect of SPD severity on neutral mental state recognition was close to zero. Conversely, Fig. 1SB suggests that the association of PCL:YV Affective scores with correctly identifying neutral mental states is significantly negative when the severity of SPD is virtually absent (SPD severity < 0.44). However, with increasing SPD severity, this association reverses (i.e., becomes positive) and reaches significance when SPD severity scores exceed 5.39.

**
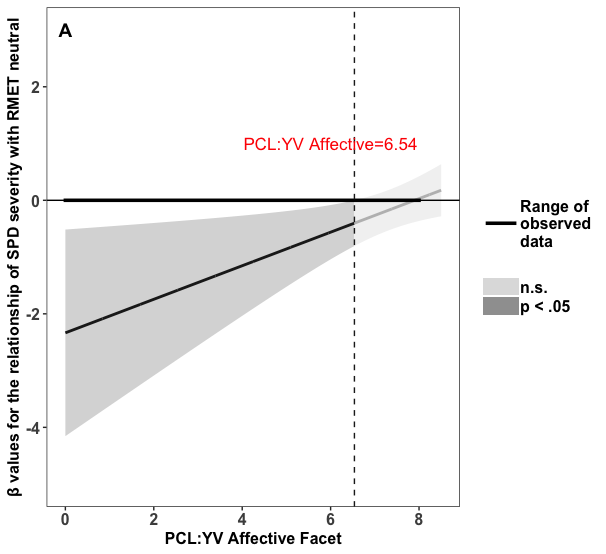

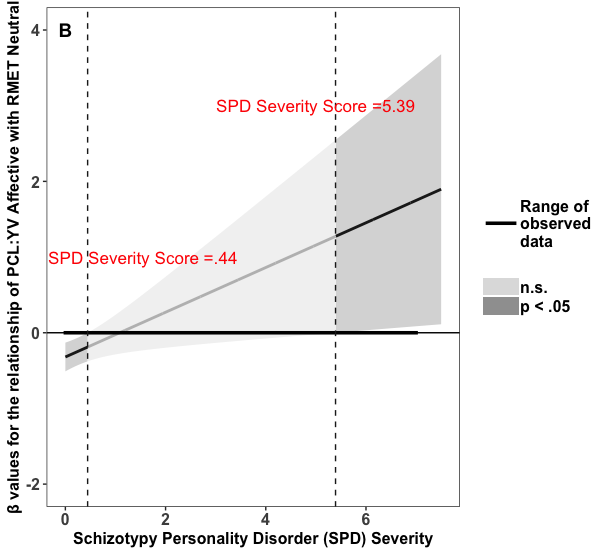
**

**Fig. 1S** The interactive association of schizotypal personality disorder (SPD) severity and Psychopathy Checklist: Youth Version (PCL:YV) Affective facet scores with RMET neutral subscale scores. Panel A displays the association (β weights) of SPD severity with participants’ performance on the neutral subscale of the RMET along the range of the PCL-YV Affective scores. The β values for the association of SPD severity with RMET neutral become less negative with increasing scores on the Affective facet of the PCL:YV, and cease to be significant when the PCL:YV Affective scores exceed 6.54 (vertical dotted line). Panel B displays the association (β weights) of PCL:YV Affective scores with participants’ performance on the neutral subscale of the RMET along the range of SPD severity scores. The significant negative β values (when SPD Severity < 0.44) for the association of PCL:YV Affective scores with RMET neutral become positive with increasing SPD severity, and significant when SPD severity scores exceed 5.39. Shaded areas represent the 95% Confidence Interval of the slopes lines (β weights). Dark grey areas represent the zone of significant associations (p < .05). Light grey areas represent the zone of non-significant associations.

The probe of the positive PCL:YV Antisocial x SPD severity interaction revealed that the negative association of SPD severity scores with correctly identifying neutral mental states reduces with increasing PCL:YV Antisocial facet scores, and is arrested when PCL:YV Antisocial scores exceed 6.82 (see Fig. 2SA). Thus, for participants whose PCL:YV Antisocial scores exceeded 6.82, the impairing effect of SPD severity on neutral mental state recognition was close to zero. In addition, Fig. 2SB suggests that the association of PCL:YV Antisocial scores on correctly identifying neutral mental states is significantly negative only when the severity of SPD is virtually absent (SPD severity < 0.29).


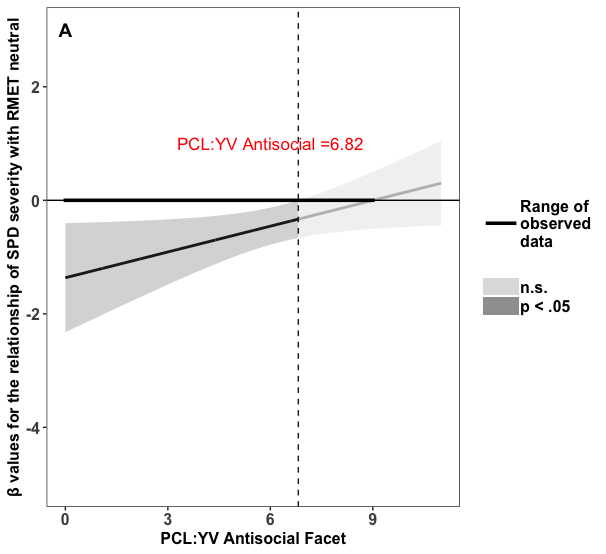

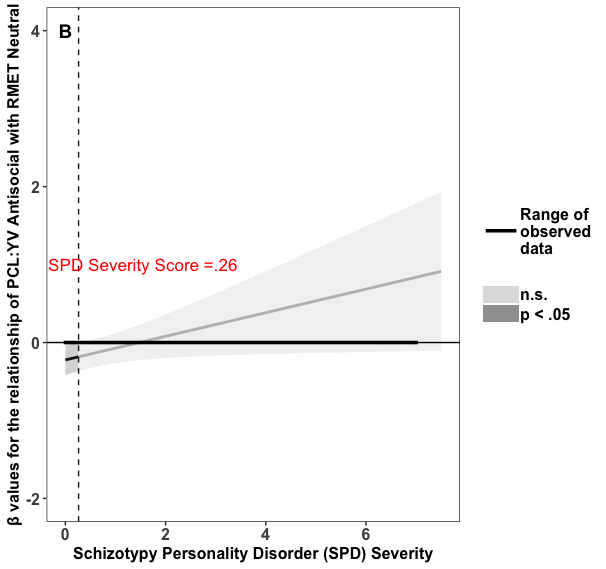


**Fig. 2S** The interactive association of schizotypal personality disorder (SPD) severity and Psychopathy Checklist: Youth Version (PCL:YV) Antisocial facet scores with RMET neutral subscale scores. Panel A displays the associations (β weights) of SPD severity with participants’ performance on the neutral subscale of the RMET along the range of the PCL-YV Antisocial scores. The β values for the association of SPD severity with RMET neutral become less negative with increasing PCL:YV Antisocial facet scores, and cease to be significant when the PCL:YV Antisocial scores exceed 6.82 (vertical dotted line). Panel B displays the association (β weights) of PCL:YV Antisocial scores with participants’ performance on the neutral subscale of the RMET along the range of SPD severity. The β values for the association of PCL:YV Antisocial scores with RMET neutral cease to be significant when SPD severity scores exceed 0.26. Shaded areas represent the 95% Confidence Interval of the slopes lines (β weights). Dark grey areas represent the zone of significant associations (p < .05). Light grey areas represent the zone of non-significant associations.
